# Pre-operative T-stage discrimination in gallbladder cancer using machine learning and DeepSeek-R1

**DOI:** 10.3389/fonc.2025.1613462

**Published:** 2025-08-01

**Authors:** Joongwon Chae, Zhenyu Wang, Duanpo Wu, Lian Zhang, Alexander Tuzikov, Magrupov Talat Madiyevich, Min Xu, Dongmei Yu, Peiwu Qin

**Affiliations:** ^1^ Institute of Biopharmaceutical and Health Engineering, Shenzhen International Graduate School, Tsinghua University, Shenzhen, Guangdong, China; ^2^ School of Communication Engineering and the Artificial Intelligence Institute, Hangzhou Dianzi University, Hangzhou, Zhejiang, China; ^3^ The First Hospital of Hebei Medical University, Shijiazhuang, Hebei, China; ^4^ United Institute of Informatics Problems, National Academy of Sciences of Belarus, Minsk, Belarus; ^5^ Department of Biomedical Engineering & Tashkent State Technical University, Tashkent, Uzbekistan; ^6^ Affiliated Fifth Hospital, Wenzhou Medical University, Wenzhou, Zhejiang, China

**Keywords:** gallbladder cancer, GBC, machine learning, large language model, DeepSeek-R1, staging, biomarker, radiology report

## Abstract

**Background:**

Gallbladder cancer (GBC) frequently exhibits non-specific early symptoms, delaying diagnosis. This study (i) assessed whether routine blood biomarkers can distinguish early T stages via machine learning and (ii) compared the T-stage discrimination performance of a large language model (DeepSeek-R1) when supplied with (a) radiology-report text alone versus (b) radiology-report text plus blood-biomarker values.

**Methods:**

We retrospectively analyzed 232 pathologically confirmed GBC patients treated at Lishui Central Hospital between 2023 and 2024 (T1, *n* = 51; T2, *n* = 181). Seven blood variables—neutrophil-to-lymphocyte ratio (NLR), monocyte-to-lymphocyte ratio (MLR), platelet-tolymphocyte ratio (PLR), carcino-embryonic antigen (CEA), carbohydrate antigen 19-9 (CA19-9), carbohydrate antigen 125 (CA125), and alpha-fetoprotein (AFP)—were used to train Random forest, Support Vector Machine (SVC), XGBoost, and LightGBM models. Synthetic Minority Over-sampling Technique (SMOTE) was applied only to the training folds in one setting and omitted in another. Model performance was evaluated on an independent test set (*N* = 47) by the area under the receiver-operating-characteristic curve (AUROC, 95% CI by 1 000-sample bootstrap confidence interval, CI); cross-validation (CV) accuracy served as a supplementary metric. DeepSeek-R1 was prompted in a zero-shot, chain-of-thought manner to classify T1 versus T2 using (a) the radiology report alone or (b) the report plus the patient’s biomarker profile.

**Results:**

Biomarker-based machine-learning models yielded uniformly poor T-stage discrimination. Without SMOTE, individual models such as XGBoost achieved an AUROC of 0.508 on the independent test set, while recall for the T1 class remained low (e.g., 14.3% for some models), indicating performance near random chance. Applying SMOTE to the training data produced statistically significant gains in cross-validation (CV) accuracy for several models (e.g., XGBoost CV Acc. 0.71 → 0.80, *p* = 0.005; LGBM CV Acc. [*No-SMOTE*] → [*SMOTE*], *p* = 0.004). However, these improvements did not translate to better discrimination on the independent test set; for instance, XGBoost’s AUROC decreased from 0.508 to 0.473 after SMOTE application. Overall, the biomarker models failed to provide clinically meaningful T-stage differentiation. DeepSeek-R1 analyzing radiology text alone reached 89.6% accuracy on the full 232-patient cohort dataset, and consistently flagged T2 cases on phrases such as “gallbladder wall thickening.” Supplying biomarker values did not change accuracy (89.6%)

**Conclusions:**

The evaluated blood biomarkers did *not* independently aid early T-stage discrimination, and SMOTE offered no meaningful performance gain. Conversely, a radiologytext-driven large language model delivered high accuracy with interpretable rationale, highlighting its potential to guide surgical strategy in GBC. Prospective multi-center studies with larger cohorts are warranted to confirm these findings.

## 1 Introduction

Gallbladder cancer (GBC) is the most common malignancy of the biliary tract, accounting for 80–95% of biliary neoplasms worldwide (1, 2). Its overall prognosis is dismal: in advanced disease the 5-year survival rate is under 5% (3, 4). This grim outlook is driven chiefly by the difficulty of early diagnosis. Early-stage GBC is almost asymptomatic, and its clinical manifestations are easily mistaken for benign gallbladder disorders. Although modern imaging techniques—ultrasonography, computed tomography (CT), and magnetic resonance imaging (MRI)—are widely used, the radiologic appearance of malignant lesions often mimics benign conditions such as xanthogranulomatous cholecystitis (XGC) (2, 5). Radiologists therefore resort to ambiguous wording in their reports, making a definitive imaging diagnosis elusive. Consequently, many patients are first recognized intra-operatively or post-operatively, and at presentation are frequently at an unresectable stage. In fact, curative surgery performed at an early stage remains the only intervention that offers a meaningful survival benefit (3). The extent of this surgery, however, is critically dependent on the pathological T-stage, which dictates the depth of tumor invasion. According to established clinical guidelines, treatment for early-stage GBC ranges from simple cholecystectomy for T1a tumors to more extensive procedures for deeper invasion. Specifically, the distinction between T1 and T2 stages is pivotal for surgical planning. While a simple cholecystectomy may suffice for some T1 tumors, T2 disease, which involves invasion into the perimuscular connective tissue, often necessitates an extended cholecystectomy including hepatectomy to improve survival outcomes (6). Inaccurate pre-operative staging can therefore lead to either undertreatment, increasing the risk of recurrence, or overtreatment, exposing patients to unnecessary surgical morbidity. Developing tools that enable earlier and more accurate pre-operative T-stage discrimination is thus critical to improving outcomes for patients with GBC.

Artificial-intelligence (AI) techniques have shown considerable promise in medicine, particularly in oncologic imaging. Machine-learning and deep-learning models are routinely employed to enhance the sensitivity and specificity of image interpretation (7). For example, a convolutional neural network trained on CT scans achieved an AUC of roughly 0.81 for distinguishing malignant from benign gallbladder lesions (4). Predictive models that couple radiomics with machine learning have also been applied to the pre-operative assessment of GBC—for instance, in predicting lymph-node metastasis—with reported AUCs of 0.82–0.85 (8). Most prior studies, however, focus exclusively on *structured* imaging metrics and quantitative data, overlooking the equally valuable *unstructured* information contained in radiology-report text. The technical language and “Impression” sections of such reports embed rich experiential judgments and subtle clues that may signal early malignancy, yet conventional ML pipelines that require structured inputs cannot exploit this resource.

Recent advances in large language models (LLMs) offer a new avenue for mining unstructured clinical text. BioGPT, for instance, achieved 78.2% accuracy on PubMedQA, demonstrating LLM potential in medical question answering (9), while Med-Gemini reached 91.2% accuracy, opening the door to LLM-supported imaging diagnosis (10). LLMs can extract disease cues from radiology reports and, in some tasks, rival or surpass human interpretation. In a pancreatic-cancer study, GPT-4 attained roughly 75% accuracy in identifying disease presence from reports, approaching the performance of clinical oncologists (11). Similarly, for automatic tumor staging, GPT-4 achieved 52–87% accuracy in assigning TNM stage for lung cancer (12). Yet, in the domain of early GBC diagnosis, there is a paucity of work that (i) quantifies the stand-alone performance of LLMs on radiology-report text, (ii) benchmarks this against traditional biomarker-based machine-learning models, and (iii) explores any incremental value that structured biomarker data may confer when combined with an LLM.

The present study addresses these gaps by independently evaluating structured biomarker-based machine learning models and an LLM that analyses unstructured radiology-report text for computational T-stage discrimination in GBC. We trained Random forest, XGBoost, LightGBM, and other algorithms on routine blood biomarkers to assess their staging capability, and separately employed the open-source LLM DeepSeek-R1 to parse radiology reports, infer lesion characteristics, malignancy likelihood, and T stage. DeepSeek-R1 employs a mixture-of-experts architecture and exhibits strong reasoning capabilities, preliminary results show excellent performance on medical tasks, matching the level achieved across multiple benchmarks by ChatGPT-o1-12-27, one of the strongest publicly released models to date (13, 14). We compared the qualitative linguistic cues extracted by the LLM with the quantitative features analyzed by the machine-learning models, and we investigated whether combining the two information sources could enhance the sensitivity and accuracy of early GBC diagnosis. Finally, we analyzed feature importance within the biomarker models to gauge the relative contribution of each marker. By demonstrating automatic stage inference from unstructured radiology text, our approach offers a new perspective for early GBC diagnosis and provides a reference point for future AI-assisted clinical decision systems.

## 2 Data collection

### 2.1 Study design and ethical approval

This retrospective study enrolled patients who underwent imaging for suspected gallbladder lesions at a single center (Lishui Central Hospital) between June 2023 and June 2024. Among the initially screened patients, 235 were finally diagnosed with gallbladder cancer (GBC) on the basis of cholecystectomy specimens or biopsy pathology. Because the primary aim was to improve early detection and staging of GBC, cases at advanced stages (T3 and T4)—which together accounted for<5% of the dataset and were too sparse for statistically meaningful analysis—were excluded. Consequently, 232 patients (T1, 51; T2, 181) constituted the final analysis cohort. Final staging was assigned according to post-operative findings.

The study protocol was approved by the Institutional Review Board (IRB) of Lishui Central Hospital (approval no. LCH-2025-056-01). Given its retrospective design, the IRB waived the requirement for written informed consent. All procedures complied with local regulations and institutional requirements. Under applicable national laws and institutional policies, neither written consent from participants nor from their legal guardians/relatives was required.

During data collection and handling, every radiology report was manually reviewed and stripped of direct identifiers—patient name, date of birth, study date, and medical-record number—before use. Only this anonymized dataset was analyzed by a locally deployed instance of DeepSeek-R1; no data were transmitted to external servers. The study fully conformed to relevant privacy regulations and to the ethical principles of the Declaration of Helsinki, ensuring confidentiality and data security throughout.

### 2.2 Data collection and variables

The key data fields used in this study are summarized in **Table 1**. Collected variables comprised: the full text of pre-operative imaging reports (CT, MRI, and MRCP), radiological impressions, post-operative pathological diagnoses, patient demographics (age and sex), and a panel of seven blood biomarkers (CEA, CA19-9, CA125, AFP, NLR, MLR, PLR).

**Table 1:** Summary of main data fields.

Field name	Description
Preop CT Imaging	Full text describing CT scan results
Imaging Diagnosis	Radiologist’s opinion (e.g., “suspected cancer”)
Preop MRCP Desc.	Detailed description of MRCP
MRCP Diagnosis	Final radiologist’s opinion from MRCP
Preop MRI Desc.	MRI findings (T1/T2, contrast, etc.)
Preop MRI Diag.	Final radiologist’s evaluation of MRI
Postop Path Desc.	Histopathologic examination (gold standard to confirm malignancy, infiltration, etc.)
Age	Patient age at time of surgery/diagnosis
Sex	Gender (M/F)
CEA (0–5)	Tumor marker; flagged if above 5 ng/mL
CA19-9 (*<*37)	Tumor marker CA19-9
CA125 (*<*35)	Tumor marker CA125
AFP (0–8.78)	Tumor marker AFP
NLR	Ratio of neutrophils to lymphocytes (inflammatory index)
MLR	Ratio of monocytes to lymphocytes
PLR	Ratio of platelets to lymphocytes
T	T stage (T1/T2/T3/T4, AJCC)
N	Lymph node metastasis (N0/N1, etc.)
M	Distant metastasis (M0/M1)
Stage	Overall Stage (I/II/III/IV, AJCC)

The selection of these biomarkers was guided by established literature demonstrating their clinical relevance in GBC. For instance, tumor markers such as CEA and CA19–9 are routinely used in clinical practice for monitoring GBC, although their diagnostic specificity can be limited (15). Similarly, AFP has been reported in rare cases of AFP-producing GBC (16). More recently, systemic inflammatory markers, including the neutrophil-to-lymphocyte ratio (NLR), platelet to-lymphocyte ratio (PLR), and monocyte-to-lymphocyte ratio (MLR), have been identified as strong predictors of overall survival. A comprehensive meta-analysis by Velasco et al. confirmed that elevated levels of these inflammatory indices are consistently associated with worse survival outcomes in GBC patients (17). Given this body of evidence suggesting their collective prognostic and diagnostic potential, we included all seven markers to test the hypothesis of whether their combined signal could effectively discriminate early T-stages.

Pathological T, N, and M categories and overall stage were assigned according to the 8th edition of the American Joint Committee on Cancer (AJCC) staging manual and served as supervised-learning labels. During data cleaning, T3 and T4 cases were excluded from model training because the study aimed to discriminate early stages (T1 and T2) and mid-/late-stage data were too sparse for meaningful analysis. To ensure analytical reliability and model stability, any record missing critical discriminative variables—such as T stage or CA19-9—was removed. Consequently, only the curated T1 and T2 samples were used for machine-learning modeling and large-language-model inference. Radiology reports exhibited minor format variations by date and author; however, given the robust natural-language capabilities of DeepSeek-R1, the original CT report text was supplied to the model without manual re-formatting.

## 3 Experiments

### 3.1 Machine learning predictive model construction

We focused particularly on cases for which both integrable imaging examinations (CT, MRI) and blood test data were available. Our primary objective was to build a classifier that distinguishes T1 from T2 cancer—an important distinction for early versus more advanced local disease—and to explore whether these features could help predict the progression of GBC. Detailed steps are outlined below.

#### 3.1.1 Feature extraction

We first loaded a spreadsheet containing demographic and clinical data for the 235 patients with pathologically confirmed GBC. Using Python’s pandas library, we removed extraneous whitespace in the column names and standardized them into English labels (e.g. Gender, CT_Images, Imaging_Diagnosis). Next, we extracted the T stage (T1, T2) from the final pathology reports with a custom text-parsing function. Cases reporting unusual notations (e.g. “T1a” or “T2b”) were simplified to integer values (1, 2) to align with the study’s focus on T1 and T2 stages.

Initially, the dataset comprised 235 patients with the following T-stage distribution: T1 (51), T2 (181), T3 (1), and T0/Tis (2). Consistent with earlier pre-processing, T3 and T4 cases were excluded because of their extreme scarcity (*<* 5% of the dataset, with only one T3 case identified) and because the clinical objective was early-stage detection. Likewise, the rare T0 or Tis cases were coded as 0 but excluded from model training. The final dataset therefore contained 232 patients (T1: 51, T2: 181).

Continuous variables (e.g. NLR, PLR, CA19-9, CEA) stored as strings were converted to numeric form by extracting the terminal numerical value in each cell, ensuring accurate representation of biomarker levels. For instance, CA19–9 values such as “The patient’s CA19-9 (*<* 37) value was 39.28” were parsed to 39.28 U/mL, yielding a mean of 104.12 U/mL (SD 625.99). CEA, CA125, and AFP were handled analogously, with means of 2.80 ng/mL (SD 2.62), 16.95 U/mL (SD 18.37), and 4.97 ng/mL (SD 21.20), respectively, reflecting the heterogeneous biomarker profiles of GBC patients.

Rows missing critical discriminative variables (e.g. T stage or CA19-9) were earmarked for exclusion; however, inspection of the 235 original cases revealed no such omissions, so no rows were dropped for missing key data. Data inconsistencies—such as repeated numeric entries (e.g. “35, 37” appearing in a single CA19–9 cell)—were resolved by retaining the last valid value, ensuring consistency across the dataset.

Ultimately, seven blood-based biomarkers—NLR, MLR, PLR, CEA, CA19-9, CA125, and AFP—were selected as features for machine-learning training. To improve model performance and stabilize distributions, each numerical feature was log-transformed via log(1+*x*) before being supplied to the learning algorithms.

**Figure 1:**
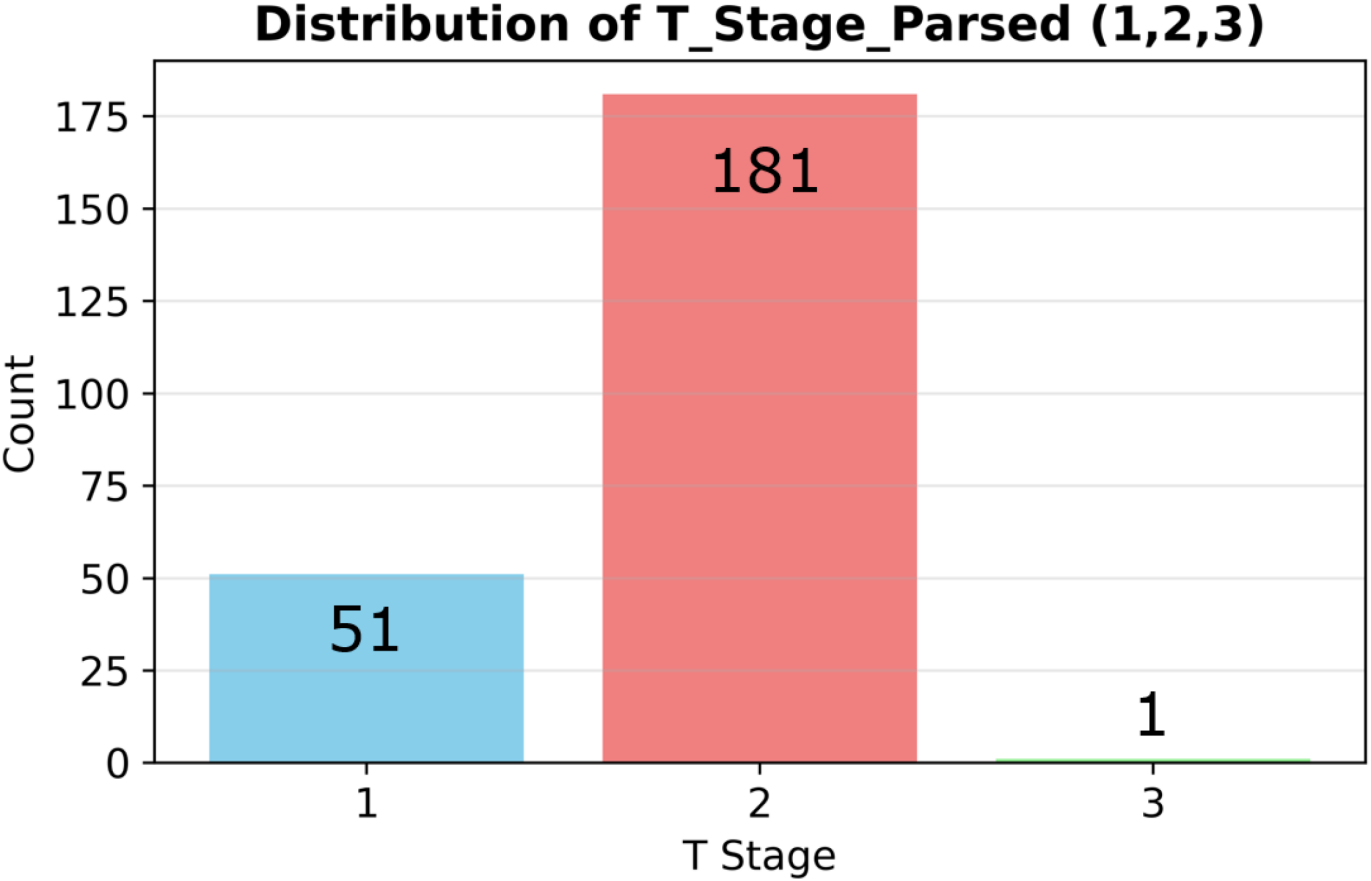
Stage distribution and biomarker histograms. Distribution of pathological T stages (T1 = 51 vs T2 = 181) in the analyzed cohort (n=232).

**Figure 2:**
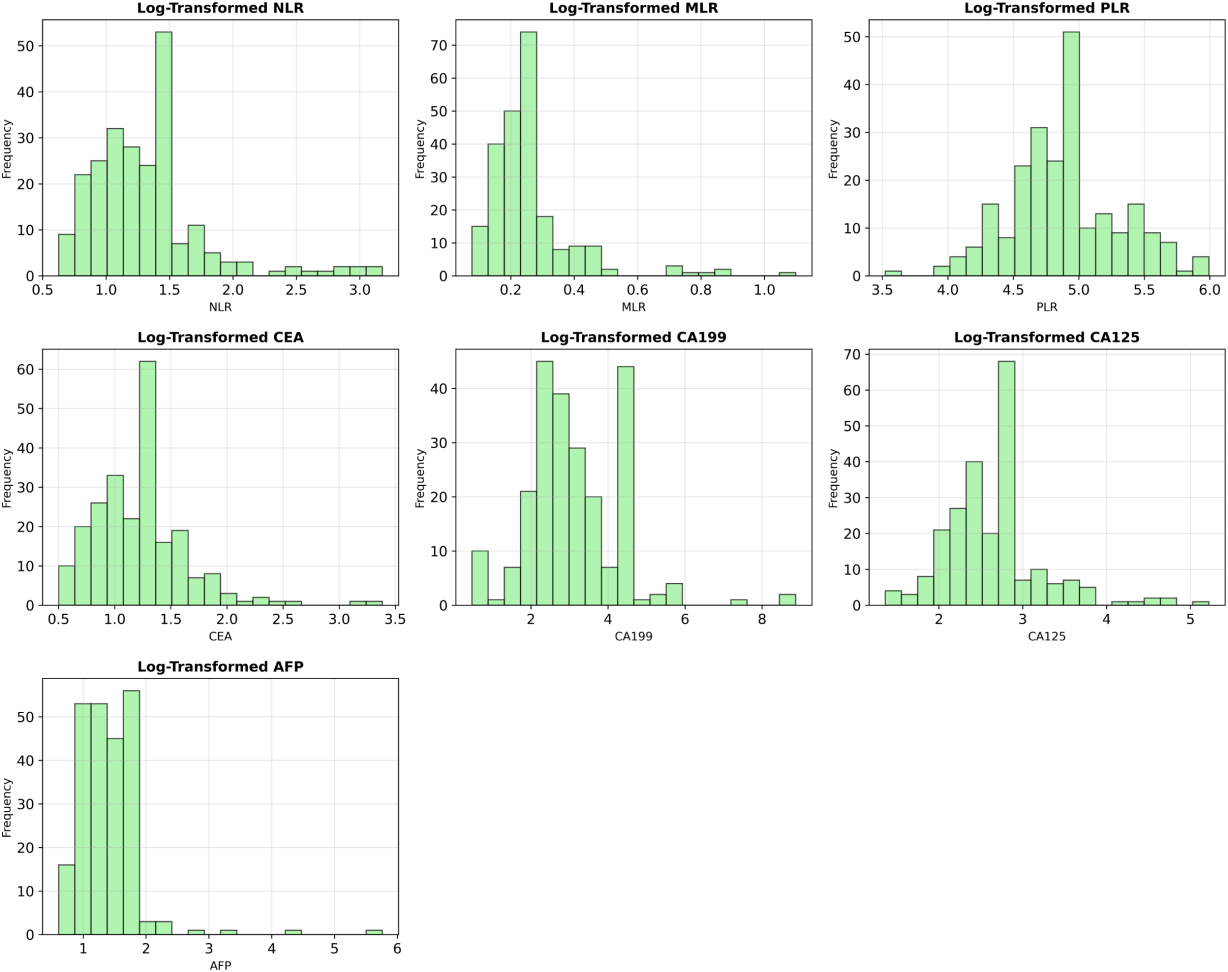
Log-transformed distributions of key biomarker variables. To mitigate the significant right-skewness present in the original data, all numerical features were log-transformed (log(1+x)), improving their distributional properties for model training.

**Figure 1** shows the post-exclusion T-stage distribution (T1 = 51 [22%], T2 = 181 [78%]). **Figure 2** plots the log-transformed biomarker histograms, where the transformation suppresses the strong right skew and stabilizes variance, allowing the models to detect the subtler differences that separate T1 from T2—an essential prerequisite for timely surgical decision-making.

#### 3.1.2 Model training and evaluation

After data cleaning, we built binary classification models to distinguish between T1 and T2 gallbladder cancer (GBC) samples. This distinction is critical for differentiating early-stage from more locally advanced GBC, directly impacting surgical planning and patient prognosis. As previously noted, T3 and T4 stage samples were excluded from this analysis due to their scarcity (less than 5% of the dataset), aligning our research focus with the clinical need for timely diagnosis of early-stage GBC.

In this study, to assess the effectiveness of addressing class imbalance, we trained and evaluated models under two distinct scenarios: one with the application of the Synthetic Minority Over-sampling Technique (SMOTE) (18), and one without. When SMOTE was applied, to prevent data leakage and ensure an objective evaluation of model performance, it was embedded within a scikit-learn Pipeline (SMOTE → StandardScaler → Classifier). This ensured that SMOTE was applied *only to the training folds* during the cross-validation process. Consequently, within each training split, the minority class (T1) was oversampled to achieve a balanced T1:T2 ratio of 1:1, while all validation folds remained strictly untouched, preserving their original data distribution for a robust assessment of generalization performance. This approach was intended to mitigate potential model bias toward the majority class (T2) and improve the identification capability for the clinically crucial minority class (T1), as correct identification of T2 stage typically necessitates extended resection, whereas T1 stage can often be managed with simple cholecystectomy.

All numerical input features (blood-based biomarkers) were standardized before model training using StandardScaler, which scales the data to have a mean of 0 and a standard deviation of 1. This step was crucial for ensuring fair performance comparisons, particularly for distance-based models like Support Vector Machine (SVC), where unscaled features could otherwise skew the results.

We trained and evaluated four primary machine learning models under each of the two scenarios (with and without SMOTE application): Random Forest (RandomForestClassifier), XGBoost (XGBClassifier), LightGBM (LGBMClassifier), and Support Vector Machine (SVC). Random Forest was selected for its robustness to noisy data and its ability to effectively handle non-linear relationships, which can be prevalent in biomarker data such as CA19–9 and NLR. XGBoost and LightGBM were chosen for their powerful gradient boosting frameworks, which are known to excel in handling imbalanced medical datasets and often provide high predictive accuracy through iterative optimization. SVC was included to explore a non-tree-based approach, leveraging its strengths in high-dimensional spaces, which is relevant given the diverse set of biomarker features in GBC.

For the Random Forest model, to further address class imbalance, it was configured with random-state=42 and the class-weight=‘balanced’ option. Hyperparameter tuning was performed using RandomizedSearchCV with 5 iterations over combinations of n-estimators (50, 100, 200), max-depth (None, 5, 10, 20), and min-samples-split (2, 5, 10) to select the best parameters for optimizing performance. The SVC model utilized the rbf kernel function with C=1.0 and gamma=‘scale’, and the probability=True option was enabled for probability estimates, with random-state=42 set for reproducibility. The XGBoost model was configured with use-label-encoder=False, eval-metric=‘logloss’, and random-state=42. LightGBM used random-state=42 as a fixed parameter to maintain consistency.

The performance of each model under each scenario (with/without SMOTE) was initially assessed on the training data using 5-fold cross-validation to calculate mean accuracy and standard deviation, providing an evaluation of model stability. Subsequently, the final performance of each model was evaluated on a separately held-out independent test set using various metrics (e.g., AUC, accuracy, precision, recall, F1-score). The detailed comparative results of model performance, including statistical significance tests for the differences observed with and without SMOTE application, are presented in the following ‘Results’ section.

### 3.2 Constructing the DeepSeek dataset and rationale for using unstructured text

To evaluate the large language model (LLM) approach, we created a text-based dataset for DeepSeek-R1. Each patient’s radiology report was combined with relevant blood test indicators (CA19-9, CEA, etc.) and assembled into a single textual input, as shown in the example below.

The narrative-form radiology reports were used in their original, unstructured format without any pre-processing, such as terminology standardization or manual keyword extraction. This approach was a deliberate methodological choice. The primary objective of this study arm was to assess the LLM’s capability to interpret real-world clinical documentation, which is inherently variable and non-standardized. By providing the raw text, we aimed to test whether an advanced LLM like DeepSeek-R1 could harness this complex, unstructured information directly, a key advantage over traditional ML models that require structured, pre-defined inputs. The reports were authored and cross-verified by three board certified radiologists specializing in GBC, ensuring a high level of clinical quality and consistency in diagnostic assessment despite stylistic variations.

“user_input”: {
“Preoperative CT”:
“The density of liver parenchyma is uniform, the intrahepatic bile duct 
is not dilated, the gallbladder is slightly enlarged, the wall is thickened, 
several stones are seen at the bottom of the gallbladder, the size is about 13.4mm, 
and the common bile duct is not dilated. The pancreas is sparse, 
with a few exudative shadows around the pancreas. 
The spleen is normal in size and uniform in density. 
There are several small round low-density shadows 
without enhancement in both kidneys, 
the largest of which is about 20.17mm, located in the left kidney, 
and no abnormalities are found in the bilateral adrenal glands. 
No lymphadenopathy is found beside the retroperitoneal aorta. 
There are no obvious abnormalities in the stomach wall. 
The distribution of the intestinal tract is normal, 
and there is no obvious thickening or edema in the intestinal wall. 
The surrounding mesenteric structure is clear, 
the bladder is full, the wall is uniform, 
and no positive stone shadows are seen in the cavity. 
There are no obvious abnormalities in the uterus and appendages. 
There are no obvious enlarged lymph nodes in the pelvic cavity. 
There are no obvious abnormalities in the appendix.
There are multiple calcifications in the arterial wall.”,
“CEA”: 1.51,
“CA19-9”: 39.28,
“CA125”: 17.07,
“AFP”: 1.99,
“NLR”: 3.454545454545454,
“MLR”: 0.36363636363636365,
“PLR”: 99.54545454545453
}

LLM Prompt Design: DeepSeek R1 was prompted with the combined textual and numerical input derived from each patient’s data. We employed and compared two primary prompting strategies to elicit TNM classification, overall stage, and explanatory reasoning:

1. Zero-shot Prompting: In this approach, the model was given direct instructions without any preceding examples. A typical zero-shot prompt was:

“You are an AI radiology assistant. Please read the following radiology report 
and blood test results for a gallbladder imaging study, then answer:
1. TNM classification: (detailed explanation for each stage)
2. Stage: (partition and rationale)
Final diagnosis: T[#] N[#] M[#], Stage [Roman numeral].”

This prompt relied on the model’s inherent understanding and its ability to follow complex instructions to generate the desired output.

2. Few-shot Prompting (with Chain-of-Thought style example): To guide the model’s reasoning process and output format more explicitly, we also utilized a few-shot prompting strategy. This involved providing the same initial instruction as the zero-shot prompt, followed by a single, illustrative example demonstrating the desired step-by-step reasoning and a complete, well-structured answer. The prompt, including the example, was as follows:

“You are an AI radiology assistant. Please read the following radiology report 
and blood test results for a gallbladder imaging study, then answer:
1. TNM classification: (detailed explanation for each stage)
2. Stage: (partition and rationale)
3. Final diagnosis: T[#] N[#] M[#], Stage [Roman numeral].”
example1:

1. TNM Classification:

T (Primary Tumor): T2

Explanation: The report describes “gallbladder is slightly enlarged, 
the wall is thickened”. Significant wall thickening is a hallmark 
of T2 disease in gallbladder cancer staging.
T2 indicates the tumor invades the perimuscular connective tissue.

N (Regional Lymph Nodes): N0

Explanation: The report explicitly states
“No lymphadenopathy is found beside the retroperitoneal aorta” and
“There are no obvious enlarged lymph nodes in the pelvic cavity.”

M (Distant Metastasis): M0

Explanation: The report describes “several small round low-density shadows 
without enhancement in both kidneys” 
consistent with benign simple cysts (common finding).

2. Stage: Stage II

Partition: T2, N0, M0.

Rationale: According to the AJCC 8th Edition staging system for gallbladder cancer:

T2N0M0 corresponds to Stage II.

3. Final Diagnosis:
T2 N0 M0, Stage II

By instructing the model to justify its answers (e.g., “detailed explanation for each stage” and “rationale” for the overall stage) under both prompting strategies, we aimed to assess not only its classification accuracy but also the plausibility and clinical relevance of its reasoning process.

Model and Inference Configuration: For all experiments, we utilized a locally deployed instance of the original DeepSeek-R1 671B model to ensure data privacy and security. DeepSeek-R1 is an open-source LLM that employs a Mixture-of-Experts (MoE) architecture, which contributes to its strong reasoning capabilities on complex tasks (13, 19). No task-specific fine-tuning was performed on any domain-specific GBC data for this study. Instead, we relied entirely on the model’s zero-shot and few-shot inference capabilities. The maximum token limit for the model’s output was set to 2,000 tokens, and all other inference parameters, such as temperature and top-p, were kept at their default values to ensure reproducibility. This setup allowed us to evaluate the model’s out-of-the-box performance in parsing and interpreting the combined unstructured medical text and structured biomarker data.

## 4 Results

This section first presents the performance of blood–biomarker–based machine-learning (ML) models for classifying T1 versus T2 gallbladder cancer (GBC), comparing results *with* and *without* the Synthetic Minority Over-sampling Technique (SMOTE). It then reports the performance of the large-language model (LLM) DeepSeek-R1, which analyses radiology reports.

### 4.1 Performance of blood-biomarker ML models

Seven biomarkers (NLR, MLR, PLR, CEA, CA19-9, CA125 and AFP) from 232 patients (T1 = 51, T2 = 181) were log-transformed, then split into a training set (185 patients; T1 = 41, T2 = 144) and an independent test set (47 patients; T1 = 10, T2 = 37).

**Figure 3:**
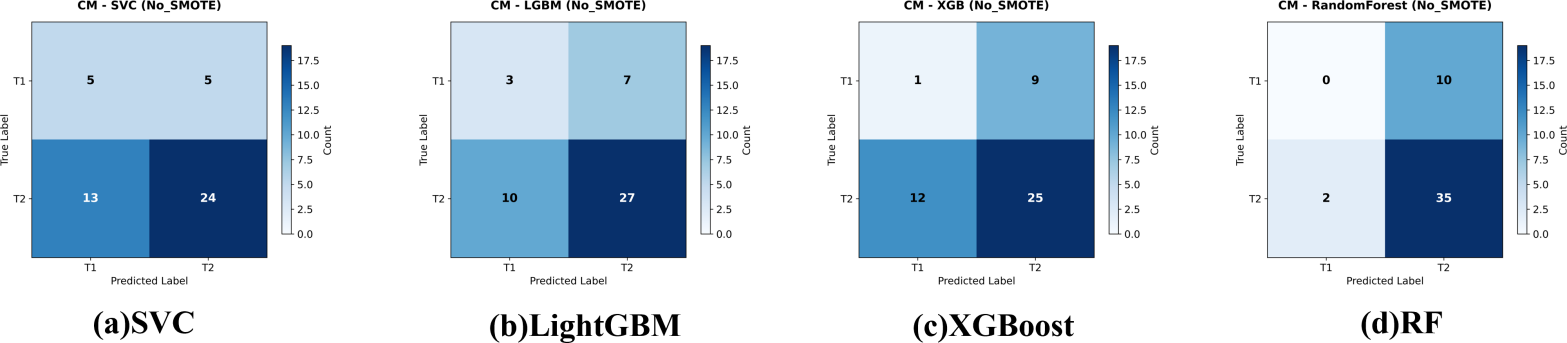
Confusion matrices for the four evaluated classifiers—**(a)** Support Vector Classifier (SVC), **(b)** LightGBM, **(c)** XGBoost, and **(d)** Random Forest—on the held-out test set for gallbladder-cancer T-stage prediction (T1 vs. T2). models give the corresponding baseline models trained on the original data. Cell values indicate the number of test cases per true-/predicted-label pair.

Four classifiers—Random Forest, SVC, XGBoost and LightGBM—were trained on the imbalanced data. Five-fold cross-validation (CV) results are given in **Table 2**. Random Forest achieved the highest mean CV accuracy (76.76 ± 3.67%), whereas SVC was the lowest (54.59 ± 6.92%). ANOVA showed significant differences among models, *F*(4,20) = 8.11*,p<* 0.001; *post-hoc* Holm-corrected tests confirmed that Random Forest significantly outperformed SVC. The confusion matrices for these baseline models are shown in **Figure 3**.

**Table 2:** Five-fold CV accuracy paired t-test (SMOTE vs. No-SMOTE) on CV accuracy.

Model	No SMOTE	SMOTE	*P*-value
Random Forest	76.76 ± 3.67	83.67 ± 6.76	0.061
SVC	54.59 ± 6.92	67.69 ± 2.59	0.007
XGBoost	70.27 ± 7.83	82.24 ± 7.68	0.005
LightGBM	66.49 ± 7.37	78.78 ± 6.08	0.004

On the independent test set (**Table 3**) all models performed poorly: AUCs clustered near random (0.38−0.54). Random Forest failed to identify any T1 cases (recall = 0%); XGBoost identified one (recall = 10%).

**Table 3:** Independent test-set performance of biomarker-only models for distinguishing T1 vs. T2 gallbladder cancer (*n* = 47).

Model	SMOTE	AUROC		AP		F1 (T1)	Recall (T1)
		Point	95% CI	Point	95% CI		
Random Forest	–	0.493	0.30-0.67	0.822	0.69-0.93	0.00	0.00
SVC	–	0.380	0.21-0.56	0.787	0.64-0.91	0.36	0.50
XGBoost	–	0.509	0.34-0.68	0.850	0.72-0.94	0.09	0.10
LightGBM	–	0.538	0.34-0.73	0.839	0.70-0.94	0.26	0.30
Random Forest	✓	0.543	0.34-0.72	0.844	0.72-0.94	0.20	0.20
SVC	✓	**0.593**	**0.43-0.76**	**0.883**	**0.78-0.95**	0.29	0.40
XGBoost	✓	0.474	0.25-0.69	0.791	0.64-0.92	0.19	0.20
LightGBM	✓	0.507	0.27-0.74	0.790	0.65-0.93	0.43	0.50

AUROC and average precision (AP) are reported with 95% confidence intervals; the last two columns report F1-score and recall for the minority class (T1).

Bold values indicate the highest performance achieved for the primary evaluation metrics (AUROC and AP).

**Figure 4:**
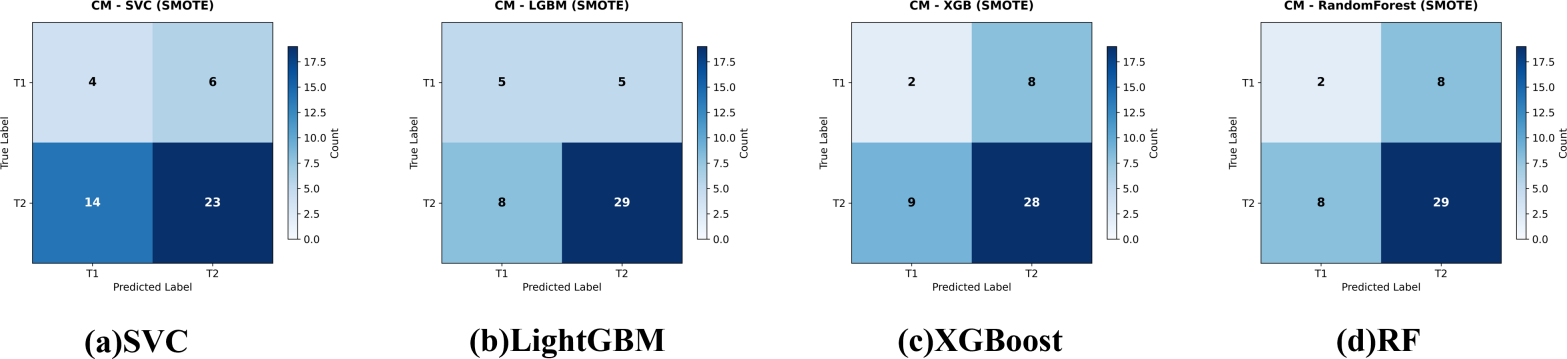
Confusion matrices for the four evaluated classifiers—**(a)** Support Vector Classifier (SVC), **(b)** LightGBM, **(c)** XGBoost, and **(d)** Random Forest—on the held-out test set for gallbladder-cancer T-stage prediction (T1 vs. T2). models trained with class-imbalance correction using SMOTE Cell values indicate the number of test cases per true-/predicted-label pair.

**Figure 5:**
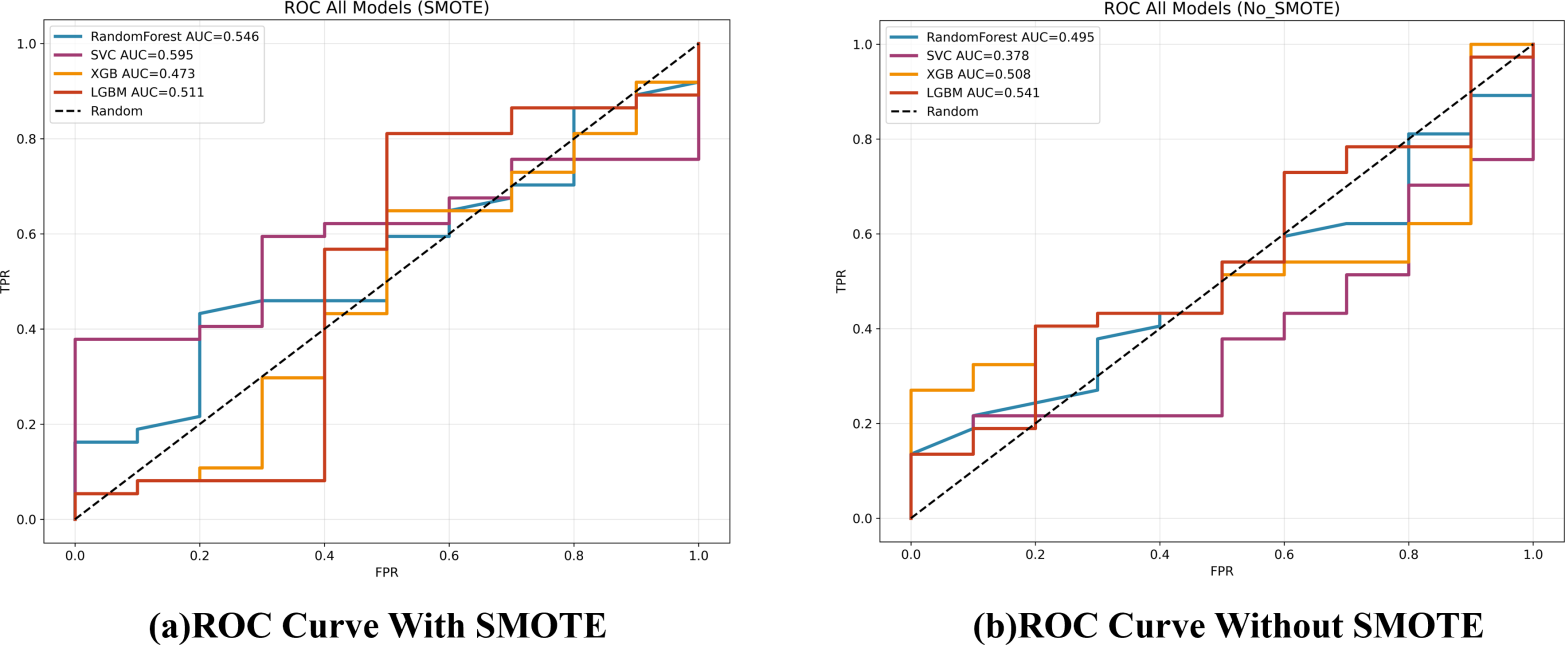
ROC curves for the four classifiers on the test set, comparing models trained with and without SMOTE.

Applying SMOTE inside each CV fold (1:1 oversampling of T1) significantly raised CV accuracy for SVC, XGBoost and LightGBM (**Table 2**); Random Forest improved but not significantly. The resulting confusion matrices, ROC curves, and a detailed statistical comparison of the AUROCs are presented in **Figures 4**, **5**, and **Table 4**, respectively.

**Table 4:** DeLong comparison of test-set AUROC (SMOTE vs. No-SMOTE).

Model	ΔAUROC	*z*	*p*
Random Forest	+0.051	-0.83	0.408
SVC	+0.216	-1.35	0.176
XGBoost	-0.035	-0.31	0.754
LightGBM	-0.030	-0.38	0.701


$$\text{AUROC}={\text{AUROC}}_{SMOTE}–{\text{AUROC}}_{No-SMOTE};\text{ none significant at }\alpha =0.05.$$

### 4.2 Correlation analysis among blood-based biomarkers

**Figure 6:**
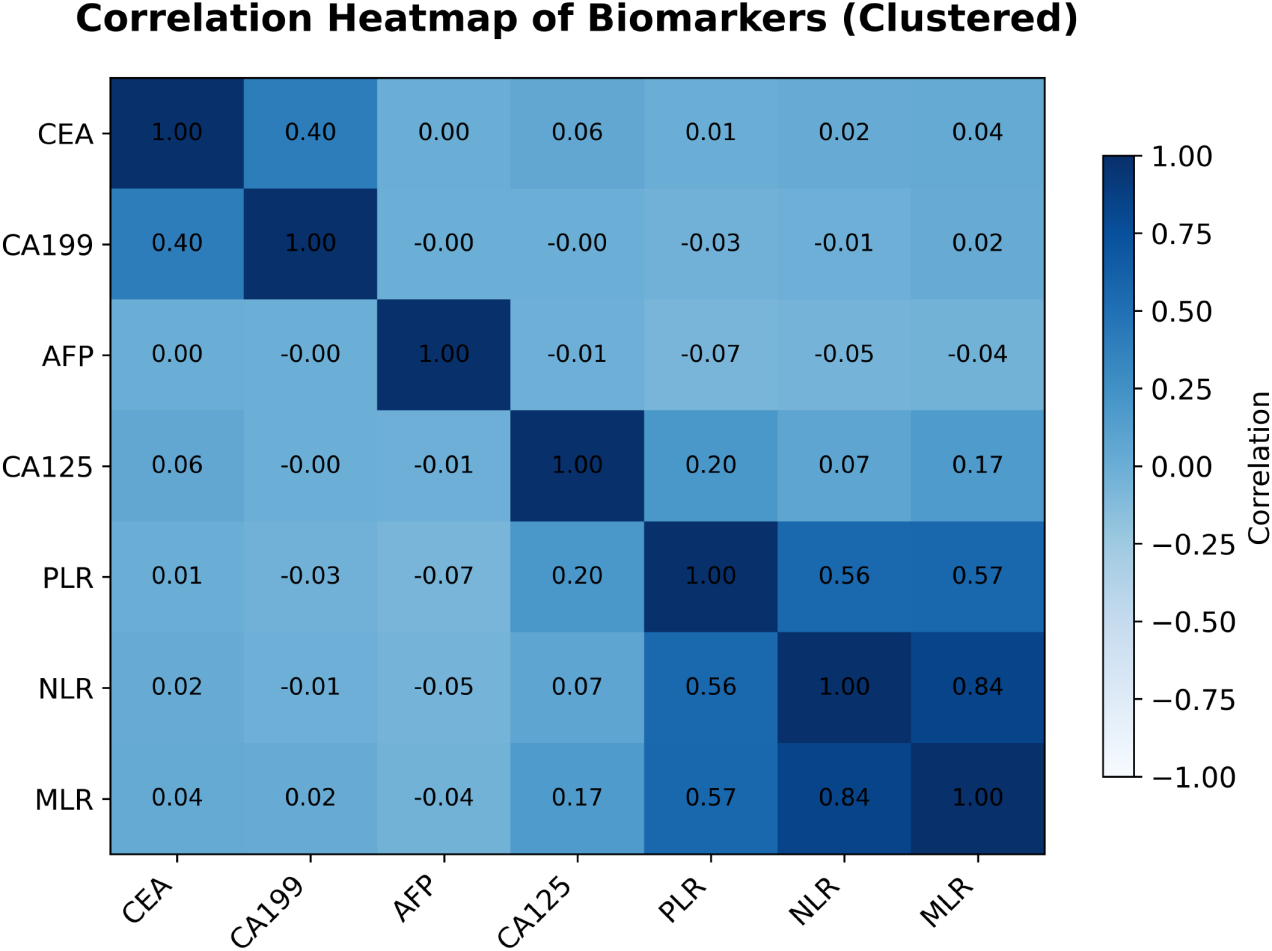
Pearson correlation heatmap of the seven blood-based biomarkers (hierarchically clustered). Numeric coefficients are shown in each cell; color intensity indicates the magnitude and direction of correlation.

To evaluate multicollinearity and inter-relationships among the seven continuous biomarkers used in the ML models—neutrophil–to–lymphocyte ratio (NLR), monocyte–to–lymphocyte ratio (MLR), platelet–to–lymphocyte ratio (PLR), carcinoembryonic antigen (CEA), carbohydrate antigen 19-9 (CA199), cancer antigen 125 (CA125) and alpha-fetoprotein (AFP)—Pearson correlation coefficients were computed and visualized as a hierarchically clustered heatmap (**Figure 6**).

The analysis exposed several notable patterns. Most striking was the strong positive correlation between NLR and MLR (*r* = 0.84). PLR correlated moderately with both NLR (*r* = 0.56) and MLR (*r* = 0.57), indicating that these inflammatory indices capture overlapping aspects of the systemic inflammatory response and may contain redundant information.

By contrast, CA125 and CA19–9 were essentially uncorrelated (*r* = −0.00). CA125 also showed weak correlations with the inflammatory ratios—NLR, MLR and PLR (*r* = 0.07, 0.17 and 0.20, respectively). A weak positive correlation was seen between CEA and CA19-9 (*r* = 0.40), whereas correlations between these tumor markers and AFP, or between the tumor markers and inflammatory indices, were generally negligible. Such low inter-marker correlations suggest that these biomarkers may represent independent biological pathways and could, in principle, provide complementary information for predictive models.

Nevertheless, as demonstrated in Sections 4.1, these statistical relationships did *not* translate into clinically useful discrimination of T1 versus T2 GBC: all trained models performed poorly. Thus, pairwise correlation structure alone is insufficient for this classification task. Future work will require more advanced feature engineering or modeling strategies capable of exploiting higher-order relationships among biomarkers.

### 4.3 Exploratory analysis of XGBoost feature attributions (SHAP values)

**Figure 7:**
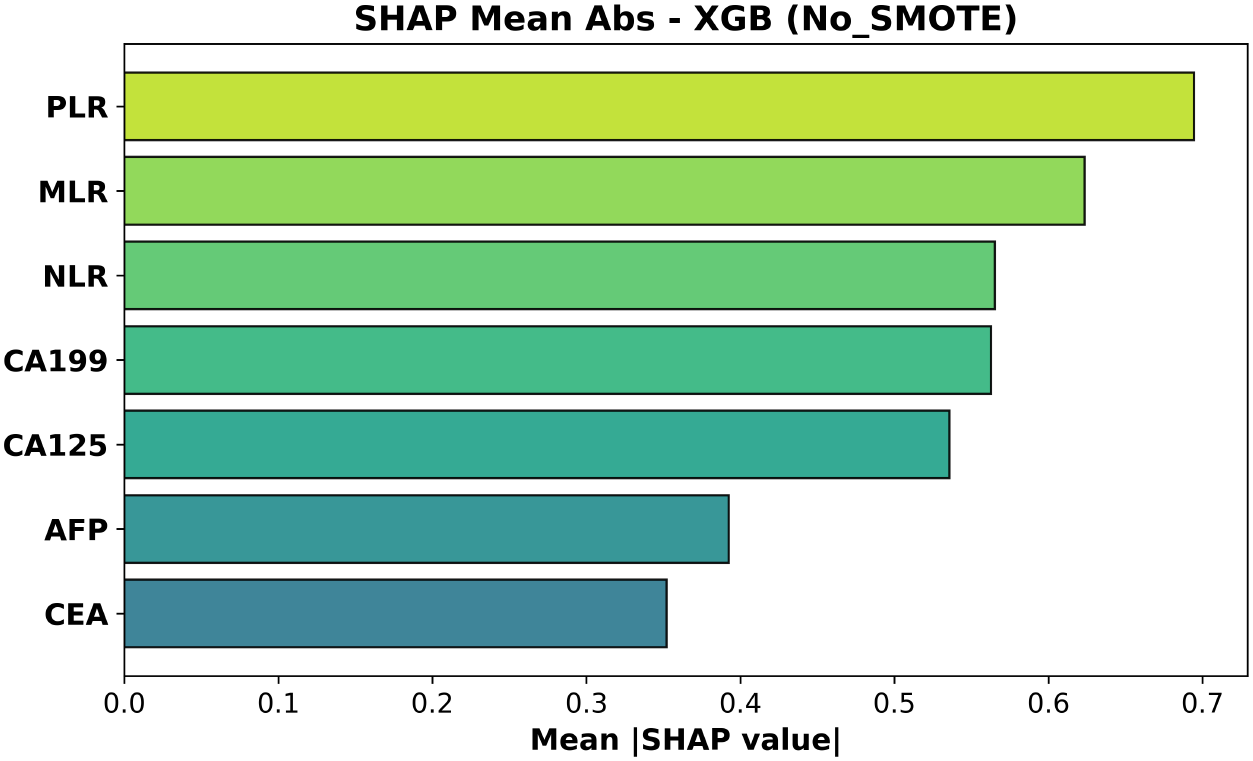
Mean absolute SHAP values for the XGBoost model trained without SMOTE. Features are ordered by average impact on the magnitude of predictions.

**Figure 8:**
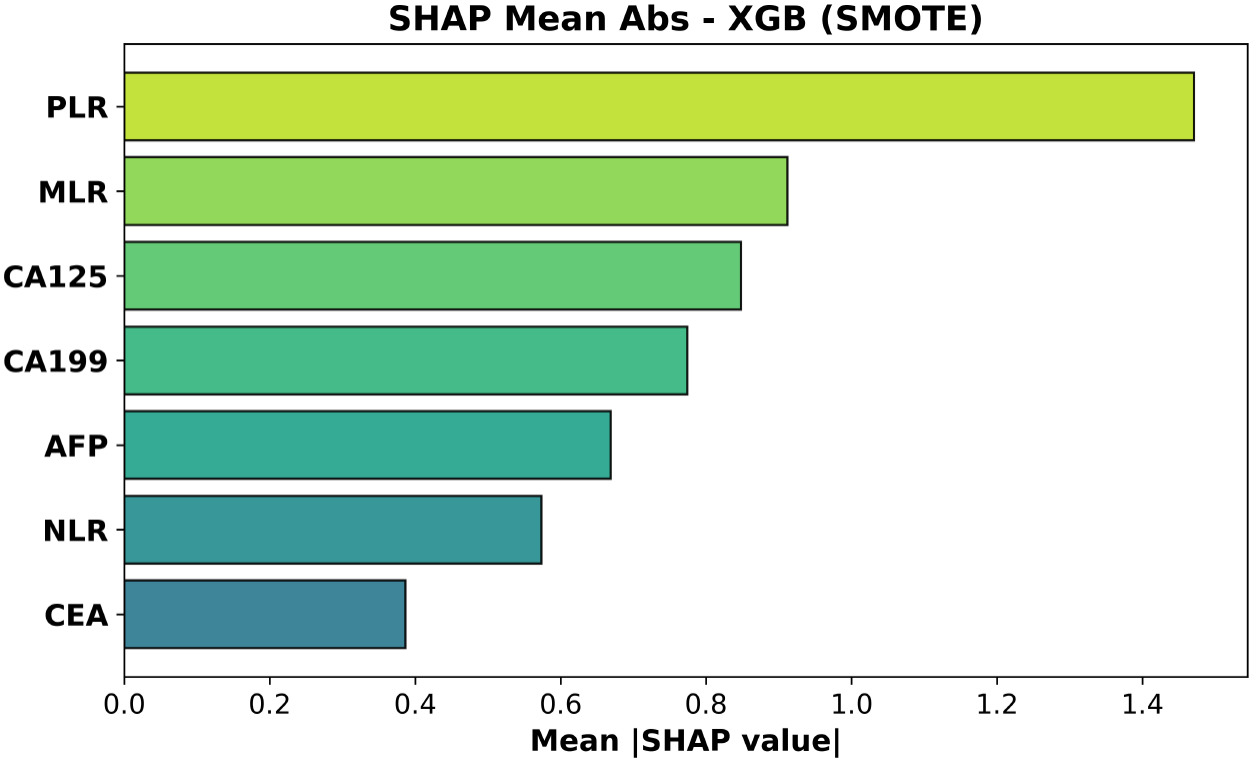
Mean absolute SHAP values for the XGBoost model trained with SMOTE. Feature ordering follows average impact on predicted probabilities.

Although the biomarker–based ML models performed poorly at distinguishing T1 from T2 GBC, we carried out an exploratory feature–attribution analysis to elucidate which variables each model *attempted* to rely on. SHAP values were computed for the XGBoost classifier trained *with* and *without* SMOTE. **Figures 7**, **8** rank features by their mean absolute SHAP value across all test-set samples.

For the SMOTE-trained model (**Figure 8**, test AUC = 0.473) platelet–to–lymphocyte ratio (PLR) exhibited the largest mean absolute impact, followed by monocyte–to–lymphocyte ratio (MLR) and CA125. Neutrophil–to–lymphocyte ratio (NLR)—often prominent in gain-based importance metrics—was less influential than PLR and MLR, while CEA contributed least.

In the non-SMOTE model (**Figure 7**, test AUC = 0.508) the top three features were again PLR, MLR and NLR; CA19–9 and CA125 had intermediate influence.

These rankings are biologically coherent: elevated *PLR* and *MLR*—and, to a lesser extent, *NLR*—capture platelet- and monocyte-driven inflammatory signaling that accompanies deeper muscular invasion (T2), whereas increases in *CA19–9* and *CA125* reflect biliary stasis or serosal irritation that become more pronounced once the tumor breaches the lamina propria. *CEA*, lacking such stage-specific behavior, accordingly shows the least contribution Wang et al. (20).

### 4.4 Illustrative model reasoning from DeepSeek-R1

The DeepSeek-R1 was evaluated for its ability to classify T-stage, N-stage, M-stage, and overall stage for gallbladder cancer (GBC) using only the radiology report text combined with relevant laboratory data, as detailed in Section 3. We primarily assessed its performance using a zero-shot prompting strategy, and also explored a few-shot prompting approach that included a chain-of-thought (CoT) style example. For the T category, DeepSeek-R1 achieved an accuracy of 89.66% on the test set, which had a skewed T1/T2 distribution (T1: 51, T2: 181). The model demonstrated a high recall for the T1 stage (0.896, see **Table 5**), indicating its effectiveness in identifying T1 cases based on textual cues. Despite the skew, the model’s ability to extract nuanced textual cues, such as “gallbladder wall thickening” or “no lymph node enlargement,” underscores the value of unstructured radiology data. By leveraging these detailed descriptors, DeepSeek-R1 achieved more accurate GBC staging than what might be obtained from structured biomarker data alone, offering a powerful tool for clinicians to refine preoperative staging and tailor surgical strategies, such as extended resections for T2 cases, ultimately improving patient outcomes in a disease where early intervention is critical.

**Table 5:** DeepSeek R1 classification metrics on the full cohort dataset.

Staging category	Accuracy (Zero-shot)	Accuracy (Few-shot + CoT)	Key observations
T-stage	89.66%	84.05%	Fewshot+CoT: biased toward T2
N-stage	100%	100%	Correctly identified all cases as N0
M-stage	100%	100%	Correctly identified all cases as M0
Overall Stage	94.40%	85.78%	Fewshot+CoT biased toward Stage II

Although the ultimate output from DeepSeek-R1 is presented in a concise format, the model’s internal reasoning can be conceptualized in several steps. Below, we describe how DeepSeek-R1 parses key CT findings, lab data, and staging criteria before arriving at its final TNM classification.

#### 4.4.1 Reasoning overview

- Gallbladder Wall Thickening and Stones. DeepSeek R1 notes that the CT report mentions “the gallbladder is slightly enlarged” with a “thickened wall” and stones at the gallbladder’s base. It recognizes that wall thickening could be consistent with malignancy if other indicators align.
- No Liver or Adjacent Organ Invasion. The report states no obvious infiltration into adjacent organs, thereby ruling out T3 or T4 classification. This is reflected in the model’s moderate to high recall for T=1 (T2 in some pathological contexts).
- Lymph Node Assessment. No suspicious lymphadenopathy is observed, suggesting N0. Notably, DeepSeek R1 achieved 100% accuracy in predicting N status based on the radiology narrative describing “no enlarged lymph nodes.”
- Distant Metastasis Consideration. The model finds no textual evidence of metastatic lesions in distant organs, concluding M0. Similarly, M classification accuracy was 100%, as no indications of metastases were described in any of the test reports.
- Laboratory Tumor Markers. Mildly elevated CA19-9 (e.g., 39.28U/mL) is noted, but other markers remain below threshold, making advanced disease less likely. While these markers help guide the model’s inferences, their primary value is in supporting or refuting extensive disease spread.
- Integrating Clinical Data. DeepSeek R1 infers likely T2 stage (perimuscular connective tissue invasion, no serosal breach), N0, M0, corresponding to Stage II. Although some Stage II samples were misclassified (leading to a Stage accuracy of 94.40%), the model generally aligned well with ground truth pathology.

#### 4.4.2 Final output from DeepSeek-R1

Below is a typical structured output from the model, demonstrating how it justifies each TNM element and arrives at the final Stage classification:

1. TNM Classification:

- T (Tumor): T2

Explanation: “The gallbladder wall is thickened”, suggesting tumor invasion into perimuscular connective tissue without evidence of serosal penetration or liver involvement.

- N (Nodes): N0

Explanation: No lymphadenopathy observed in the retroperitoneum or pelvic regions, indicating no regional lymph node metastasis.

- M (Metastasis): M0

Explanation: No distant metastases detected in other organs or nonregional lymph nodes.

2. Staging:

- Stage IIA (T2N0M0)

Judgment Criteria: The tumor invades the perimuscular connective tissue but remains confined to the gallbladder, with no nodal or distant spread.

3. Final diagnosis: T2N0M0, Stage II

In this example, the LLM’s textual explanation correlates imaging findings with standard TNM definitions, effectively reproducing reasoning akin to clinical decision making. Despite mild elevation of CA19-9, the absence of overt metastatic evidence or liver invasion leads to a Stage II determination. Indeed, DeepSeek R1 achieved an overall Stage classification accuracy of 94.40%, while achieving perfect scores (100%) for both N and M classification. For T, it exhibited an accuracy of 89.66%. While the dataset had a skewed distribution toward T2 cases, the model effectively utilized textual information to achieve this level of accuracy. These results underscore the value of unstructured radiology data, and how an LLM can harness such data to achieve more accurate GBC staging than what might be obtained from structured biomarker data alone.

## 5 Discussion

This study evaluated the performance of two independent AI approaches for pre-operative T-stage discrimination in pathologically confirmed gallbladder cancer (GBC). The first approach trained several machine-learning (ML) models—including Random Forest and XGBoost—on seven blood-based biomarkers (NLR, MLR, PLR, CEA, CA19-9, CA125, AFP). Across the independent test set, these models achieved only modest discrimination, with maximum AUCs of roughly 0.55 (no SMOTE) to 0.60 (SMOTE-applied SVC). These results suggest an intrinsic limitation of the chosen biomarker panel in reliably detecting the subtle differences between T1 and T2 GBC. This finding aligns with previous research, which has also indicated that while such biomarkers may hold prognostic value, their utility for precise pre-operative staging, particularly in distinguishing early stages, is limited (17, 21).

Although SMOTE significantly increased cross-validation (CV) accuracy (e.g., XGBoost, *p* = 0.005), it failed to improve—and sometimes reduced—test-set performance (XGBoost AUC 0.508 → 0.473). This apparent contradiction can be explained by the mechanics of oversampling. Techniques like SMOTE can improve the classification decision boundary, thus boosting threshold-dependent metrics like accuracy.

However, by generating synthetic minority samples, they can also distort the original probability distribution and the rank-ordering of instances, which negatively impacts rank-based metrics like AUROC (22). This phenomenon has been reported to be common in small, imbalanced datasets, and the characteristics of our cohort likely contributed to this outcome.

By contrast, the second approach—text analysis of radiology reports using the open-source LLM DeepSeek-R1—yielded highly encouraging results. With zero-shot prompting alone, the model achieved 89.6% accuracy for T-stage and 100% accuracy for both N- and M-stage classification. This demonstrates that narrative cues such as “gallbladder wall thickening” or “no enlarged lymph nodes” embed decisive information for staging and that an LLM can exploit these cues without explicit feature engineering.

Interestingly, a few-shot prompt containing a Chain-of-Thought (CoT) example *decreased* T-stage accuracy to 84.05%. This aligns with prior work showing that CoT or few-shot examples do not always improve—and can even degrade—LLM performance on complex medical tasks (23, 24). Confirmation-bias phenomena reported by Turpin et al. (25) may have contributed (25). Hence, direct zero-shot querying may outperform CoT in certain domains, underscoring the need for systematic prompt-design research. In the few-shot+CoT configuration, the model produced 195 correct predictions out of the 232 test cases; among the 37 errors, 17 involved an over-staging of true T1 lesions to T2, whereas the remaining 20 were erroneously labeled as the non-existent “T3” category. In contrast, under zero-shot prompting the model misclassified 24 cases: four true T1 lesions were upgraded to T2, and the other 20 were likewise assigned to T3.

Clinically, LLM-based report analysis could assist surgeons in refining pre-operative plans (e.g., deciding on extended resection for T2 lesions). Our findings parallel those of Chen et al. (11), who demonstrated LLM utility for pancreatic-cancer reports (11). Nevertheless, LLM performance inevitably depends on the radiologist’s descriptive quality and terminology. Future systems must progress from text-only interpretation to direct analysis of raw imaging (CT, MRI) so that AI can bypass or supplement human subjectivity.

This study analyzed the two AI pipelines separately, multimodal fusion of structured biomarkers, unstructured text, and imaging features was beyond scope. Developing such integrative models will likely be pivotal for further gains in GBC diagnosis.

### 5.1 Limitations

This study has several limitations that should be acknowledged.

1. Single-center, retrospective cohort and generalizability: All 232 cases were drawn from a single hospital (Lishui Central Hospital), which limits the external validity and generalizability of our findings. The performance of our models, particularly the LLM, may not translate directly to other institutions with different reporting styles or patient populations. Multi-center prospective studies are required to confirm our results.
2. Class imbalance and its impact on model performance: Our dataset exhibited a significant class imbalance between T1 (n=51) and T2 (n=181) cases, and advanced stages (T3/T4) were too rare for meaningful analysis. This imbalance severely hindered the ML models’ ability to predict the minority class, a challenge that even a technique like SMOTE could not overcome, as evidenced by the poor test-set performance.
3. Limited discriminative power of the selected biomarker panel: The seven-marker panel, while based on established prognostic factors, produced AUCs below 0.60. This confirms that these systemic markers, despite their value in predicting overall survival, lack the specificity required to distinguish the subtle local invasion differences between early T-stages. Future work should explore more extensive or novel biomarker panels.
4. LLM dependence on report quality and prompt engineering: The high accuracy of DeepSeek-R1 is contingent on the quality and detail of the radiologists’ narrative reports. Performance may vary significantly with different terminology, levels of descriptive detail, or report structures. Furthermore, as our results showed a performance drop with few-shot prompting, the model’s output is also sensitive to prompt design, highlighting the need for systematic prompt engineering research.
5. No direct image analysis: This study relied solely on textual reports and did not incorporate direct analysis of raw imaging data (e.g., CT/MRI scans). A truly multimodal AI system that integrates visual features from images with textual and biomarker data would likely be more robust and represents a critical direction for future research.

Addressing these limitations will require multi-center prospective data, enhanced biomarker panels, prompt optimization, and truly multimodal AI that unifies image analysis and natural-language reasoning.

## 6 Conclusion

This proof-of-concept study compared two artificial-intelligence pipelines for pre-operative T-stage assessment in gallbladder cancer (GBC): (i) a machine-learning (ML) model trained on seven routine blood biomarkers, and (ii) the open-source large language model (LLM) DeepSeek-R1 applied directly to radiology-report text. In an independent test cohort of 47 patients (T1 = 10, T2 = 37) the biomarker-based ML models achieved area-under-the-curve (AUC) values below 0.60, confirming limited clinical utility. By contrast, DeepSeek-R1, without any task-specific fine-tuning, reached a T-stage accuracy of 89.6% and classified N- and M-stage with 100% accuracy, demonstrating that linguistic cues in narrative reports encode rich staging information.

These findings suggest that automated LLM analysis could become a valuable decision-support tool—particularly for borderline lesions where the need for extended resection hinges on reliable T2 detection. Nevertheless, the present approach remains constrained by (i) its single-center, retrospective design, (ii) dependence on the radiologist’s terminology and reporting style, and (iii) the absence of direct image interpretation by the LLM.

Future work should therefore pursue multi-center prospective validation, integrate raw CT/MRI analysis with textual and biomarker inputs in a truly multimodal architecture, and address regulatory pathways for clinical deployment. Such next-generation systems have the potential to improve both diagnostic accuracy and surgical planning precision in GBC.

## Data Availability

The dataset includes the patient’s age, blood tests, CT test results and postoperative progress. No data will be provided separately. Now that we have disclosed the name of the hospital and the duration of the data collection, we believe that disclosure of this sensitive data risks specifying the patient’s information. Requests to access the datasets should be directed to JC, cai-zy24@mails.tsinghua.edu.cn.

## References

[1] Hundal R Shaffer EA. Gallbladder cancer: epidemiology and outcome. Clin Epidemiol. (2014) 6:99–109. doi: 10.2147/CLEP.S37357, PMID: 24634588 PMC3952897

[2] Roa JC García P Kapoor VK Maithel SK Javle M Koshiol J. Gallbladder cancer. Nat Rev Dis Primers. (2022) 8:69. doi: 10.1038/s41572-022-00398-y, PMID: 36302789 PMC12314663

[3] Gupta P Basu S Arora C. Applications of artificial intelligence in biliary tract cancers. Indian J Gastroenterol. (2024) 43:1–12. doi: 10.1007/s12664-024-01518-0, PMID: 38427281

[4] Yin Y Yakar D Slangen JJ Hoogwater FJ Kwee TC de Haas RJ. The value of deep learning in gallbladder lesion characterization. Diagnostics. (2023) 13:704. doi: 10.3390/diagnostics13040704, PMID: 36832192 PMC9954814

[5] Fu T Bao Y Zhong Z Gao Z Ye T Zhang C. Machine learning-based diagnostic model for preoperative differentiation between xanthogranulomatous cholecystitis and gallbladder carcinoma: a multicenter retrospective cohort study. Front Oncol. (2024) 14:1355927. doi: 10.3389/fonc.2024.1355927, PMID: 38476361 PMC10927717

[6] Miao W Liu F Guo Y Zhang R Wang Y Xu J. Research progress on prognostic factors of gallbladder carcinoma. J Cancer Res Clin Oncol. (2024) 150:447. doi: 10.1007/s00432-024-05975-0, PMID: 39369366 PMC11456552

[7] Bhardwaj V Sharma A Parambath SV Gul I Zhang X Lobie PE. Machine learning for endometrial cancer prediction and prognostication. Front Oncol. (2022) 12:852746. doi: 10.3389/fonc.2022.852746, PMID: 35965548 PMC9365068

[8] Liu X Liang X Ruan L Yan S. A clinical-radiomics nomogram for preoperative prediction of lymph node metastasis in gallbladder cancer. Front Oncol. (2021) 11:633852. doi: 10.3389/fonc.2021.633852, PMID: 34631512 PMC8493033

[9] Luo R Sun L Xia Y Qin T Zhang S Poon H. Biogpt: generative pre-trained transformer for biomedical text generation and mining. Briefings Bioinf. (2022) 23:bbac409. doi: 10.1093/bib/bbac409, PMID: 36156661

[10] Saab K Tu T Weng W-H Tanno R Stutz D Wulczyn E. Capabilities of gemini models in medicine. arXiv preprint arXiv:2404.18416. (2024).

[11] Chen L-C Zack T Demirci A Sushil M Miao B Kasap C. Assessing large language models for oncology data inference from radiology reports. JCO Clin Cancer Inf. (2024) 8:e2400126. doi: 10.1200/CCI.24.00126, PMID: 39661914

[12] Nakamura Y Kikuchi T Yamagishi Y Hanaoka S Nakao T Miki S. Chatgpt for automating lung cancer staging: feasibility study on open radiology report dataset. medRxiv. (2023), 2023–12. doi: 10.1101/2023.12.11.23299107

[13] Liu A Feng B Xue B Wang B Wu B Lu C. Deepseek-v3 technical report. arXiv preprint arXiv:2412.19437. (2024).

[14] Faray de Paiva L Luijten G Puladi B Egger J. How does deepseek-r1 perform on usmle? medRxiv. (2025) 2025–02. doi: 10.1101/2025.02.06.25321749

[15] Sachan A Saluja SS Nekarakanti PK Nimisha Mahajan B Nag HH. Raised ca19–9 and cea have prognostic relevance in gallbladder carcinoma. BMC Cancer. (2020) 20:1–8. doi: 10.1186/s12885-020-07334-x, PMID: 32867709 PMC7457344

[16] Laurent MS Esterl RM Jr. Halff GA Speeg KV. Gallbladder carcinoma producing alpha-fetoprotein. J Clin Gastroenterol. (1999) 28:155–8. doi: 10.1097/00004836-199903000-00015, PMID: 10078826

[17] Velasco R Tan H Juan M. Haematologic biomarkers and survival in gallbladder cancer: a systematic review and meta-analysis. ecancermedicalscience. (2024) 18:1660. doi: 10.3332/ecancer.2024.1660, PMID: 38425767 PMC10901636

[18] Chawla NV Bowyer KW Hall LO Kegelmeyer WP. Smote: synthetic minority over-sampling technique. J Artif Intell Res. (2002) 16:321–57. doi: 10.1613/jair.953

[19] Guo D Yang D Zhang H Song J Zhang R Xu R. Deepseek-r1: Incentivizing reasoning capability in llms via reinforcement learning. arXiv preprint arXiv:2501.12948. (2025).

[20] Wang Y-F Feng F-L Zhao X-H Ye Z-X Zeng H-P Li Z. Combined detection tumor markers for diagnosis and prognosis of gallbladder cancer. World J Gastroenterol: WJG. (2014) 20:4085. doi: 10.3748/wjg.v20.i14.4085, PMID: 24744600 PMC3983467

[21] Kang JS Hong SY Han Y Sohn HJ Lee M Kang YH. Limits of serum carcinoembryonic antigen and carbohydrate antigen 19–9 as the diagnosis of gallbladder cancer. Ann Surg Treat Res. (2021) 101:266–73. doi: 10.4174/astr.2021.101.5.266, PMID: 34796142 PMC8564080

[22] Blagus R Lusa L. Smote for high-dimensional class-imbalanced data. BMC Bioinf. (2013) 14:1–16. doi: 10.1186/1471-2105-14-106, PMID: 23522326 PMC3648438

[23] Singhal K Azizi S Tu T Mahdavi SS Wei J Chung HW. Large language models encode clinical knowledge. Nature. (2023) 620:172–80. doi: 10.1038/s41586-023-06291-2, PMID: 37438534 PMC10396962

[24] Patel D Raut G Zimlichman E Cheetirala SN Nadkarni G Glicksberg BS. The limits of prompt engineering in medical problem-solving: a comparative analysis with chatgpt on calculation based usmle medical questions. MedRxiv. (2023), 2023–08. doi: 10.1101/2023.08.06.23293710

[25] Turpin M Michael J Perez E Bowman S. Language models don’t always say what they think: Unfaithful explanations in chain-of-thought prompting. Adv Neural Inf Process Syst. (2023) 36:74952–65.

